# Mybl2 rejuvenates heart explant‐derived cells from aged donors after myocardial infarction

**DOI:** 10.1111/acel.13174

**Published:** 2020-06-19

**Authors:** Ghazaleh Rafatian, Maryam Kamkar, Sandrine Parent, Connor Michie, Yousef Risha, André S. D. Molgat, Richard Seymour, Erik J. Suuronen, Darryl R. Davis

**Affiliations:** ^1^ Department of Cellular and Molecular Medicine University of Ottawa Ottawa ON Canada; ^2^ Division of Cardiology University of Ottawa Heart Institute Ottawa ON Canada; ^3^ Division of Cardiac Surgery University of Ottawa Heart Institute Ottawa ON Canada

**Keywords:** aging, gene therapy, ischemic cardiomyopathy, Mybl2, rejuvenation, stem cells and regenerative medicine

## Abstract

While cell therapy is emerging as a promising option for patients with ischemic cardiomyopathy (ICM), the influence of advanced donor age and a history of ischemic injury on the reparative performance of these cells are not well defined. As such, intrinsic changes that result from advanced donor age and ischemia are explored in hopes of identifying a molecular candidate capable of restoring the lost reparative potency of heart explant‐derived cells (EDCs) used in cell therapy. EDCs were cultured from myocardial biopsies obtained from young or old mice 4 weeks after randomization to experimental myocardial infarction or no intervention. Advanced donor age reduces cell yield while increasing cell senescence and the secretion of senescence‐associated cytokines. A history of ischemic injury magnifies these effects as cells are more senescent and have lower antioxidant reserves. Consistent with these effects, intramyocardial injection of EDCs from aged ischemic donors provided less cell‐mediated cardiac repair. A transcriptome comparison of ICM EDCs shows aging modifies many of the pathways responsible for effective cell cycle control and DNA damage/repair. Over‐expression of the barely explored antisenescent transcription factor, Mybl2, in EDCs from aged ICM donors reduces cell senescence while conferring salutary effects on antioxidant activity and paracrine production. In vivo, we observed an increase in cell retention and vasculogenesis after treatment with Mybl2‐over‐expressing EDCs which improved heart function in infarcted recipient hearts. In conclusion, Mybl2 over‐expression rejuvenates senescent EDCs sourced from aged ICM donors to confer cell‐mediated effects comparable to cells from young nonischemic donors.

## Graphical abstract

Aging modifies some of the canonical pathways responsible for effective cell cycle control in aged ischemic heart derived cells. Mybl2 rejuvenates these heart explant‐derived stem cells from aged mice. As such, after transplantation into the infarcted hearts, the rejuvenated cells convey therapeutic effects similar to the cells derived from young healthy mice.

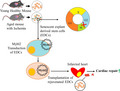

## 1 INTRODUCTION

Aging is the most important risk factor for cardiovascular disease (Lloyd‐Jones et al., 2009). While a quarter of the healthy young adult heart is composed of cardiomyocytes, aging results in progressive myocyte loss with compensatory hypertrophy in surviving tissue (Olivetti et al., 1995). Given that the incidence of myocardial infarction is also greatest in the elderly, it is unsurprising that many suffer from ischemic cardiomyopathy (ICM; Lloyd‐Jones et al., 2009) and that these patients have become the focus for new innovative approaches, such as cell therapy.

Of the numerous cell products tested, heart‐derived cells have garnered much attention with favorable Phase 1 and 2 studies demonstrating safety and possible efficacy (Ishigami et al., 2015, 2017; Makkar et al., 2012; Tarui et al., 2015). These cells of intrinsic cardiac origin (White et al., 2013) have been shown to provide a potent repertoire of cytokines and extracellular vesicles (EVs) that stimulate endogenous repair to reduce ventricular scarring and promote the generation of new blood vessels and myocytes (Chimenti et al., 2010; Ibrahim, Cheng, & Marban, 2014; Kanda & Davis, 2017). In contrast to the controversial c‐Kit+ literature, the mechanistic evidence supporting heart‐derived therapeutics is quite persuasive with 45+ independent laboratories confirming improvements in heart function when delivered after injury (Davis, 2019). While recent work suggests that aging fundamentally impacts the biology of heart‐derived cells by increasing markers of DNA damage and senescence, the interplay between donor age and heart function remains to be clarified, as all reports have used cells from patients with multiple medical comorbidities that cloud straightforward interpretation of study conclusions (Cheng et al., 2014; Mishra et al., 2011; Nakamura et al., 2016).

As such, we explored the intrinsic changes resulting from advanced donor age and a history of ischemic injury in hopes of identifying a molecular candidate capable of rejuvenating heart explant‐derived cells (EDCs). To isolate the direct effects of donor status on cell function and to avoid the effects of prolonged culture, we focused on the primary outgrowth from plated cardiac samples (EDCs; Davis et al., 2010; Mayfield et al., 2016; Molgat et al., 2014) rather than expanded cardiosphere‐derived cells or antigenically selected subpopulations. Similar to cardiosphere‐derived stem cells, EDCs provide a therapeutically relevant collection of CD105+ cells that contain endothelial and mesenchymal progenitor subpopulations without evidence for hematopoietic contamination (Davis et al., 2009).

Transcriptome profiling of EDCs has revealed changes in pathways related to cell cycle control and DNA repair system as well as a reduction in Mybl2 expression with advanced donor age and a history of ischemia. This finding rationalized efforts to explore whether Mybl2, a critical regulator of cell cycle progression and senescence (Joaquin & Watson, 2003), was capable of rejuvenating aged ischemic EDCs to a youthful pro‐healing phenotype. Mybl2 was specifically selected due to its key roles in the expression of genes related to DNA replication and checkpoint control pathways and maintenance of genome stability (Heinrichs et al., 2013; Mowla, Lam, & Jat, 2014). Overall, we identified Mybl2 as a prototypical antisenescent molecule capable of rejuvenating aged ischemic EDCs to a youthful phenotype and thus achieved higher therapeutic efficacy.

## 2 RESULTS

### 2.1 Aging and ischemic remodeling combine to impair cell function

**Figure 1:**
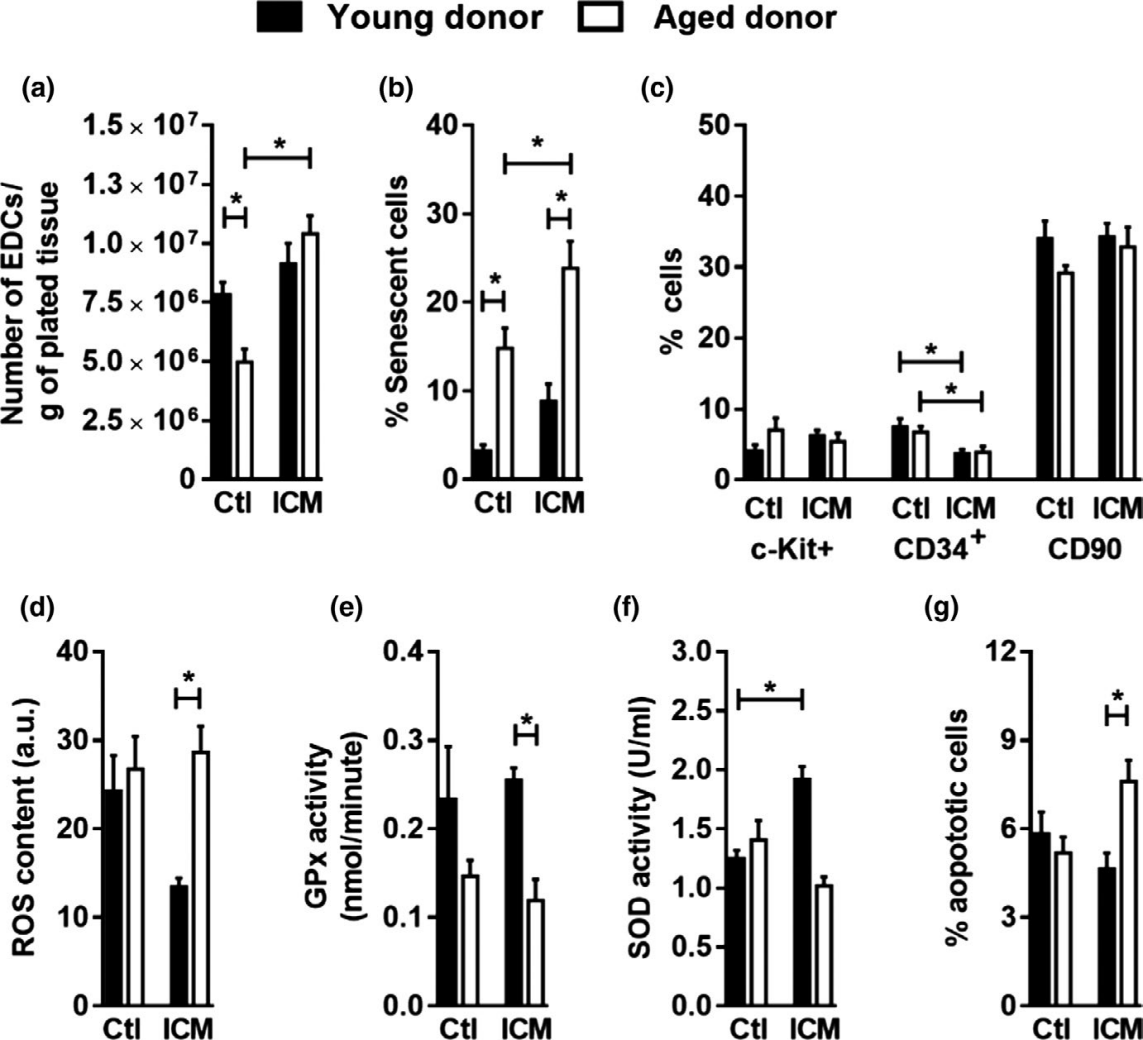
Effects of donor age and ischemia on EDCs. (a) The total number of cells collected from 1 g of cultured heart tissue (*n* = 15). (b) Senescent cell quantification by senescence‐associated β‐galactosidase staining (*n* = 8). (c) Flow cytometry demonstrating the percentage of c‐Kit+, CD34+, and CD90+ cells found within EDCs (*n* = 8). (d) Fluorometric measure of ROS content within EDCs (*n* = 5). (e) Glutathione peroxidase (GPx) activity (*n* = 5) and (f) superoxide dismutase activity within EDCs (*n* = 5). (g) Flow cytometric evaluation of Annexin V+/7‐AAD+EDCs after 48 hr of culture in 1% oxygen 1% serum stress conditions (*n* = 7). Values are mean ± *SEM*. *p ≤ .05 as indicated. EDCs, explant‐derived cells; ROS, reactive oxygen species

The effect of advanced donor age on EDC cell cultures was evaluated by comparing cardiac tissue from young (8 weeks old) to aged (54 weeks old) C57 mice (control, Ctl groups). The latter representing the point beyond which C57 mice begin to exhibit age‐related changes and senescent markers (Figure S1). As shown in Figure 1a, advanced donor age resulted in 38 ± 8% fewer cells available for enzymatic collection and correlated with a greater number of cells expressing the senescent markers β‐galactosidase, caveolin‐1, lamin‐B1 and increased cell size (Figure 1b and Figure S2). These differences were not associated with EDC phenotype as age had no effect on the main EDC markers (Davis et al., 2009, 2010), CD34+, CD90+ or c‐Kit+ (Figure 1c). Age also had little effect on the ability of collected cells to handle reactive oxygen species (ROS, Figure 1d‐f) or to withstand apoptosis within a harsh ischemic environment (Figure 1g).

The effect of ischemic injury on EDCs was investigated in aged and young mice sacrificed 4 weeks after the left anterior coronary artery (LCA) ligation (ischemic, ICM groups). As shown in Figure S3 and Table S1, age did not influence the early progression of cardiac remodeling after injury (*p* = ns for effect of age on left ventricular function). Ischemic injury had only a modest effect on the number of cells expanded from the young tissue biopsies, but markedly increased cell culture yields when combined with advanced donor age (Figure 1a). Akin to aging alone, ischemic injury had little effect on the proportional make‐up of EDCs, excepting a minor ≈3% decrease in CD34+ cell content (Figure 1c). The cells cultured from ischemic injured tissue were more likely to be senescent (Figure 1b) and, when combined with increased donor age, less able to withstand apoptosis (Figure 1g); a finding that likely reflects reduced antioxidant reserves and ability to handle ROS stress (Figure 1d‐f). In contrast, cells grown from young tissue after ischemic injury responded in a very different manner with no increase in apoptosis and a markedly better ability to withstand ROS stress with the latter being attributable to increased superoxide dismutase (SOD) activity (Figure 1f). Thus, while aging alone had little effect on many of the cell autonomous parameters except for increased senescence that influence therapeutic repair, ischemic injury increased EDC apoptosis within stress conditions and senescence which reflected altered handling of ROS.

### 2.2 Advanced donor and recipient age combine with ischemia to limit cell treatment outcomes

**Figure 2:**
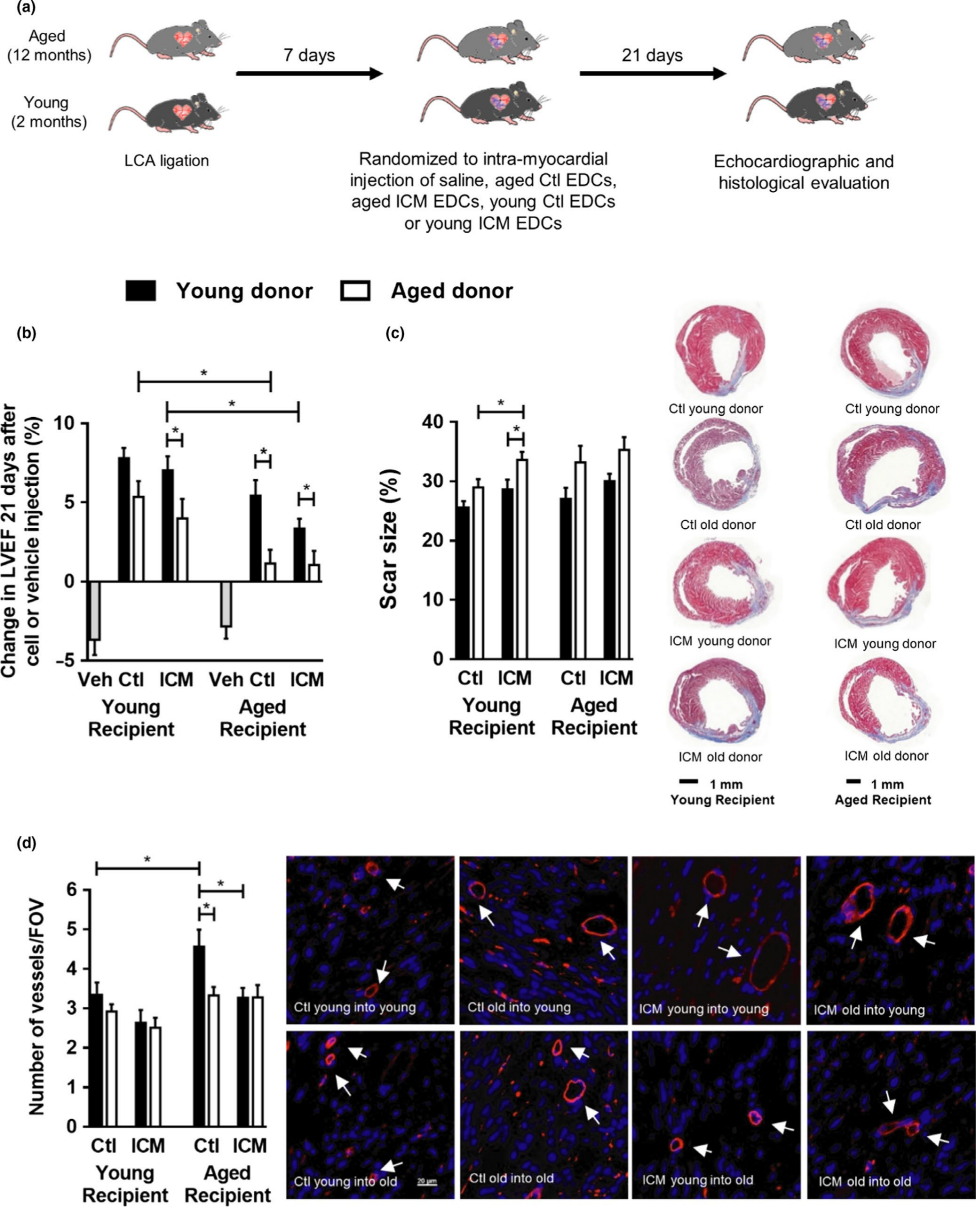
Evaluation of EDC performance in vivo. (a) A schematic overview of the experiment. (b) LVEF functional difference (∆EF %) at 3 weeks after EDC injection compared to baseline evaluation at Day 7 (*n* = 12). (c) Scar size evaluation calculated using Masson’s trichrome staining by measuring fibrotic tissue relative to the whole ventricular area on heart sections at 3 weeks post‐EDC treatment (*n* = 6). Representative images of Masson’s trichrome staining; left panel (d) The number of vessels per field of view in the infarct border zone calculated after isolectin B4 staining (*n* = 6). Representative images of isolectin B4 staining; left panel Ctl, control; ICM, ischemic; Veh, vehicle. Values are mean ± *SEM*. *p ≤ .05 as indicated. EDCs, explant‐derived cells; LVEF, left ventricular ejection fraction

Ischemic cardiomyopathy disproportionately affects older patients. We investigated the influence of age and ICM on EDC‐mediated repair of ischemic myocardium in both young (2 months) and aged (12 months) recipient mice (Figure 2a). One week after LCA ligation, all mice were randomly allocated to receive intramyocardial injection of vehicle, young Ctl EDCs, young ICM EDCs, aged Ctl EDCs or aged ICM EDCs. As shown in Figure 2b and Table S2, injection of vehicle alone resulted in progressive adverse cardiac remodeling over the following 3 weeks (*p* = .51 for effect of recipient age). Although EDCs from all cohorts significantly improved cardiac function when compared to vehicle injection alone (*p* ≤ .01), advanced recipient age attenuated the salutary effect of EDC transplantation; suggesting that the old heart milieu is much less responsive to reparative stimuli. Cells from young donors consistently outperformed those from aged donors (*p* ≤ .05 effect for age). Histological analysis confirmed that these results as injection of cells from aged donors was associated with greater myocardial scars compared to young donors (Figure 2c and Figure S4). In contrast to the effect of ICM on fibrosis expansion following transplantation of ICM donors, we observed that ICM had no significant effect on the cardiac functional improvement of transplanted cells. Interestingly, recipient age had an unanticipated effect on vessel density as aged recipients treated with young donor EDCs demonstrated greater peri‐infarct vessel densities (Figure 2d), suggesting that divergent mechanisms may underlie the observed effects.

### 2.3 Aging and cardiac remodeling alter the cytokine and extracellular vesicle profile of EDCs

**Figure 3:**
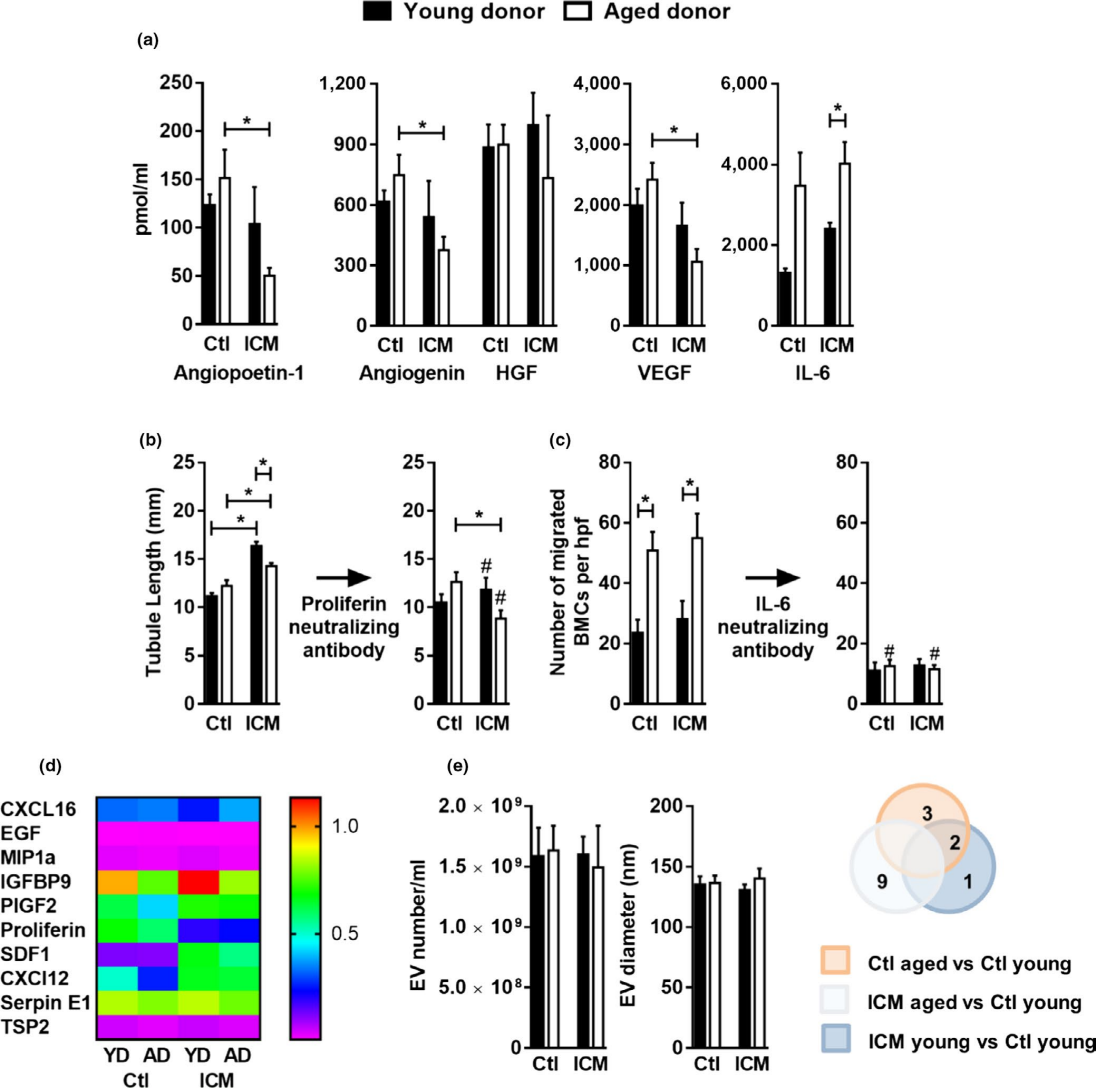
Paracrine signaling of EDCs. (a) EDC secretion of angiopoietin‐1, angiogenin and HGF, VEGF, and IL‐6 (*n* = 4). (b) Quantification of tubule formation before (left panel) and after (right panel) proliferin depletion (*n* = 4). (c) Quantification of migrated cells before (left panel) and after (right panel) IL‐6 depletion using neutralizing IL‐6 antibody (*n* = 5). (d) Heat map showing angiogenic cytokines with differential secretion levels (*n* = 3). (e) Total extracellular vesicles number and their average size collected over 48 hr (*n* = 4; left panels). The number of miRNA differentially expressed relative to control young EDCs detected by RNA sequencing (*n* = 3) Ctl, control; ICM, ischemic. Values are mean ± *SEM*. *p ≤ .05 as indicated; #*p* ≤ .05 in equivalent cohorts before versus after cytokine depletion. EDCs, explant‐derived cells

The connection between senescence of transplanted cells and postischemic heart function prompted a closer evaluation of the effects of age and ischemia on paracrine production by EDCs. A candidate screening approach targeting the most abundant cytokines commonly produced by heart‐derived cells (Latham et al., 2013; Rafatian & Davis, 2018) revealed that advanced donor age had negligible effects on angiogenin, angiopoetin‐1, hepatocyte growth factor and vascular endothelial growth factor while, akin to other medical comorbidities (Mayfield et al., 2016), markedly increased the secretion of interleukin 6 (IL‐6; Figure 3a). Our in vitro testing did not indicate an influence of advanced donor age on the ability of EDC conditioned media to stimulate new vessel growth (Figure 3b), though age was associated with a notable increase in bone marrow‐derived mononuclear cell recruitment (Figure 3c). IL‐6 neutralization abrogated this effect suggesting the observed changes in cell recruitment were likely attributable to increased production of this cytokine (Figure 3c). Ischemia, in contrast, markedly increased the pro‐angiogenic ability of EDC‐conditioned media with superior effects noted when endothelial cells were exposed to young donor conditioned media (≈1.5‐fold greater, Figure 3b). Interestingly, these effects in aged ICM donors occurred despite a marked reduction in the production of pro‐angiogenic cytokines commonly produced by EDCs (Figure 3a). To identify whether an unmeasured cytokine may be responsible for the observed differences, an unbiased comparative proteomic analysis was conducted (Figure S5), which revealed an increase in the secretion of the angiogenic placental hormone proliferin within ischemic EDC conditioned media. Antiproliferin antibody neutralization markedly reduced tubule formation which suggests a causative role in the observed differences (*p* ≤ .05 vs. nonproliferin depleted; Figure 3b,d). Ultracentrifugation was used to isolate EVs from EDC conditioned media (Figure S6). While age and ischemia had no effect on the production or size of EVs within conditioned media (Figure 3e), RNA sequencing revealed that age and ischemia altered some of the small RNAs found within EDC‐sourced EVs. EVs sourced from aged ICM donors contained large quantities of Piwi‐interacting RNAs implicated in mediating gene silencing (Table S3). Differentially expressed miRNAs found within aged ICM EVs were found to target signal transducers, receptors, and binding proteins which are known to have roles in mediating wound healing. The pattern of these miRNA alterations within aged ICM EVs resembled pathologic conditions with likely negative paracrine effects on neighboring cells (Tables S4 and S5). Analysis of EVs mRNA content showed elevated secretion of mRNA involved in splicing and ubiquitination such as Rnf123 that plays role in degradation of cell cycle cyclins (Table S5). Taken as whole, these data suggest that age and ischemia combine to have potent suppressive effect on the paracrine profile of EDCs via negative changes in the cytokine and EV signature.

### 2.4 Advanced donor age and ischemic injury alter the molecular signature of EDCs

**Figure 4:**
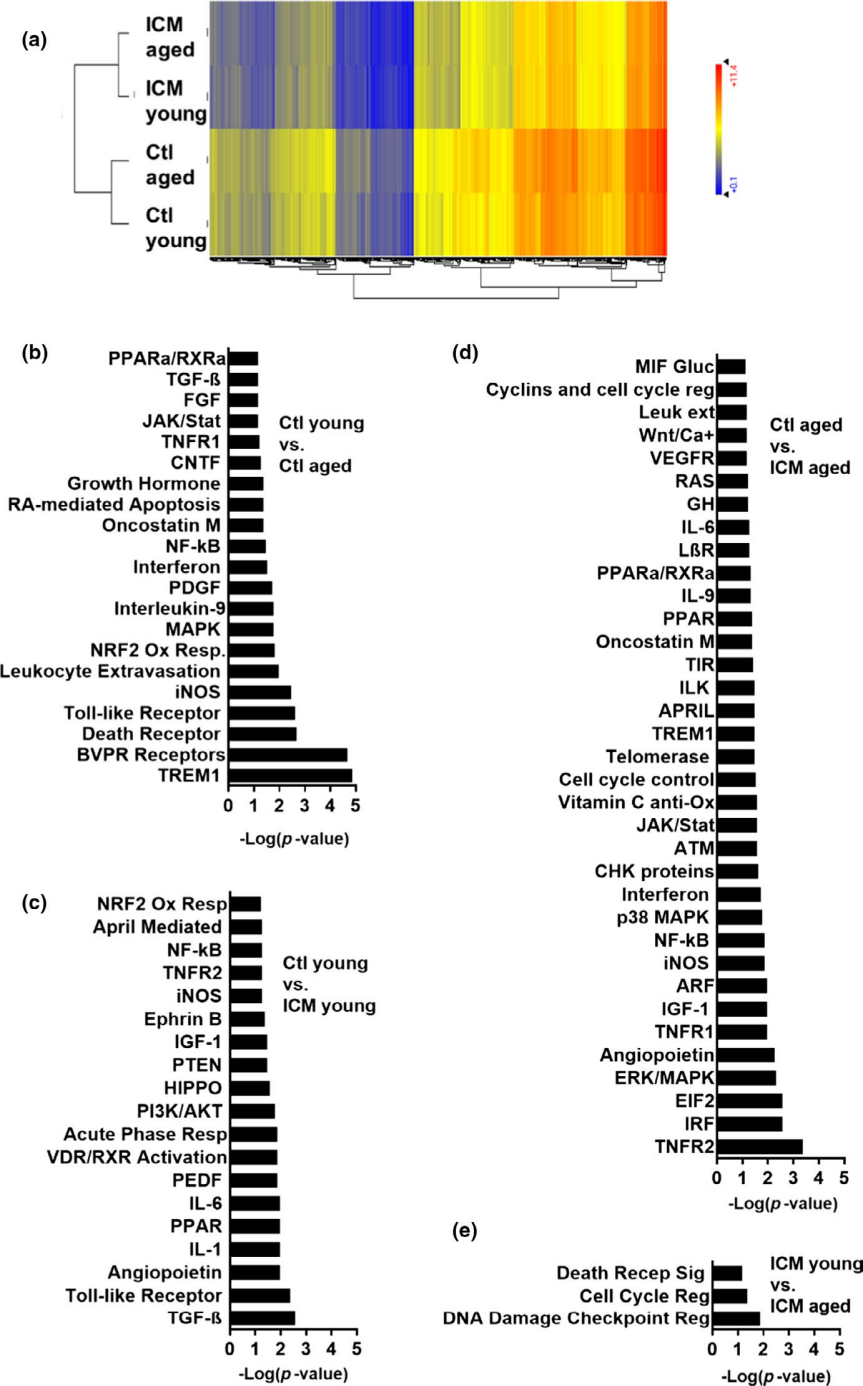
Microarray analysis (*n* = 3). (a) Hierarchical clustering of genes with more than twofold differential expression (FDR ≤ 0.05). (b) Significantly altered canonical pathways determined by ingenuity pathway analysis, based on genes whose expression was significantly different between groups (*p* ≤ .01); *x*‐axis shows significance of the changes in a pathway. Ctl, control; FDR, false discovery rate; ICM, ischemic

**Figure 5:**
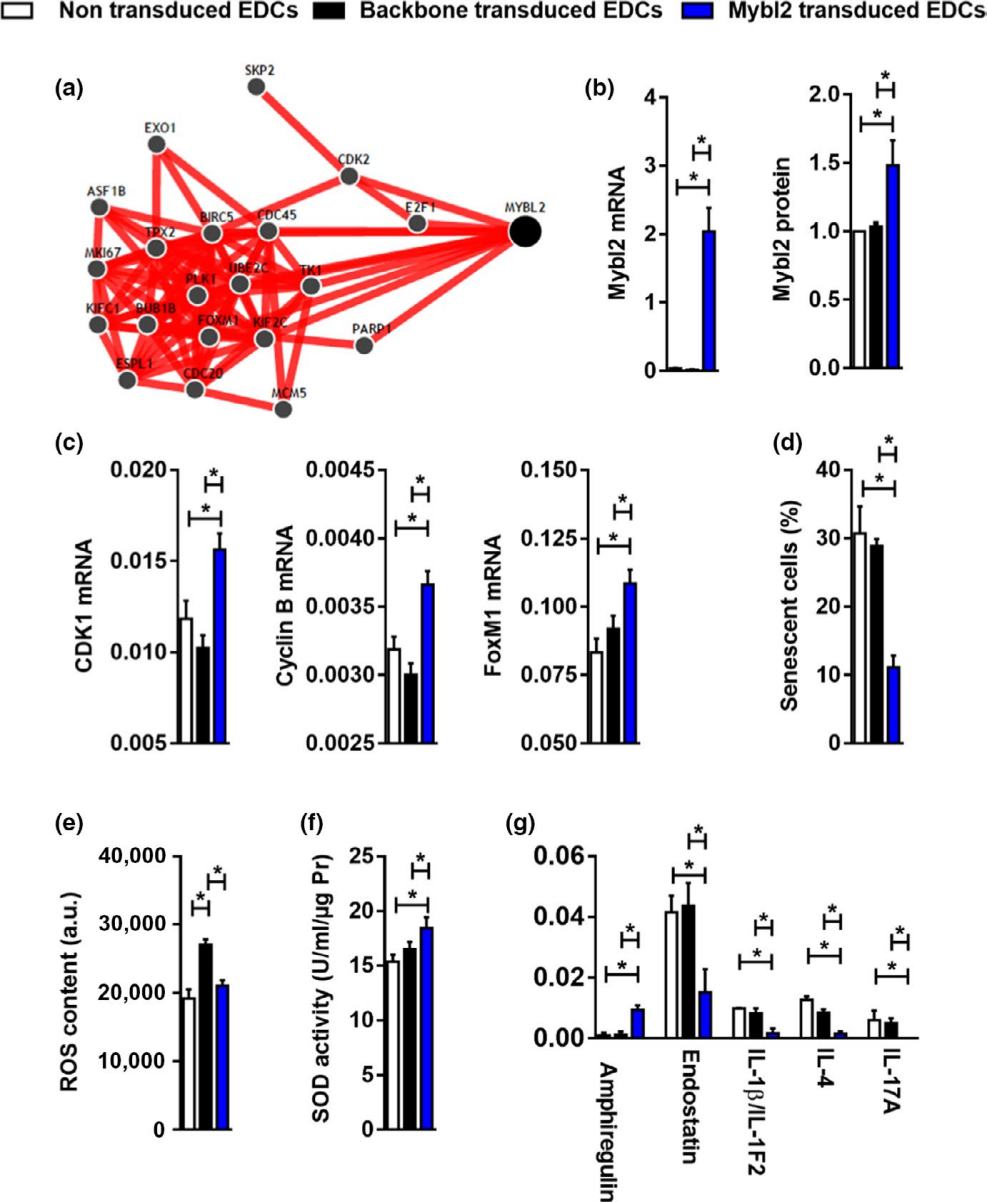
Mybl2 gene transfer effect on EDCs in vitro. (a) GIANT prediction of Mybl2 network. (b) Mybl2 expression and protein levels 48 hr after EDC transduction relative to GAPDH (*n* = 4). (c) Mybl2 over‐expression effect on its target genes Cyclin B, CDK1, and FoxM1 (*n* = 4). (d) The percentage of senescent cells following Mybl2 transduction (*n* = 9). (e) Mybl2 transduction effect on ROS content (*n* = 3) and (f) SOD activity (*n* = 4) within EDCs. (g) Five cytokines with significant differential expression was identified using profiling of mouse secreted cytokines after Mybl2 transduction. Values are mean ± *SEM*. *p ≤ .05 as indicated. EDCs, explant‐derived cells; ROS, reactive oxygen species; SOD, superoxide dismutase

To explore fundamental changes to EDCs caused by age and ischemia, we compared global gene‐expression profiles to identify transcripts underlying the observed changes in cell function and paracrine signaling. As shown in Figure 4, analysis of transcripts with a twofold or greater change (false discovery rate [FDR] ≤ 0.05) revealed that aging alone prompted the differential expression of 245 genes within pro‐inflammatory pathways that included NF‐КB, TREM1, Toll‐like receptor and death receptor signaling. Cells cultured from ICM hearts resulted in the differential expression of 924 and 773 genes in EDCs sourced from aged and young donors, respectively (≥twofold change, FDR ≤ 0.05). Within young EDCs, ICM upregulated transcripts responsible for cell migration, proliferation, and survival within the protein kinase A, NRF2‐mediated oxidative stress response, and PPAR signaling pathways. In contrast, in aged EDCs, ICM increased the expression of transcripts within pathways responsible for IL‐6, NF‐КB, TNFR2, TREM1, and p38 MAPK signaling. Most interestingly, aging promoted the differential expression of 145 genes in ICM EDCs (≥twofold change, FDR ≤ 0.05) which modify many of the canonical pathways responsible for effective cell cycle control and DNA damage/repair (cell cycle control of chromosomal replication, DNA damage induced 14‐3‐3σ signaling, mitotic roles of Polo‐like kinase and cell cycle: G2/M DNA damage checkpoint regulation). Differences between the transcriptome of aged and young donors with a history of myocardial infarction were explored in depth. The activation or inhibition of multiple upstream regulators was predicted (including TBX2, E2F1, FoxM1, EP400, RB1, HDAC1, and Let‐7; Table S6A). Based on analysis documenting dysregulation within multiple pathways underlying cell cycle regulation and survival, the data were further combed for genes with a role in cell cycle control/senescence displaying differential expression greater than 1.5‐fold (Table S6B). From several identified molecules, the Genome‐scale Integrated Analysis of gene Networks in Tissues (GIANT) database identified Mybl2 as possessing a strong association with genes related to DNA replication and cell cycle checkpoint regulation including some regulators that were predicted to have altered activity (such as E2Fs and FoxM1, Figure 5a). Confirmatory quantitative polymerase chain reaction (qPCR) analysis demonstrated an expression reduction in Mybl2 transcriptome of EDCs from aged ICM donors compared young donors (Figure S7).

### 2.5 Mybl2 rejuvenates cell function, paracrine output, and therapeutic regeneration

The possibility that altering a single transcript might revitalize EDCs from aged mice after myocardial injury was evaluated using lentiviral‐mediated somatic gene transfer. As shown in Figure 5b,c, successful over‐expression of Mybl2 increased the expression of relevant target transcripts (CDK1, cyclin B, and FoxM1) within aged ischemic EDCs. This increase translated into ≈65% fewer senescent EDCs relative to control transduced or nontransduced cells (Figure 5d). While lentiviral transduction alone significantly increased the production of toxic intermediates, Mybl2 over‐expression reduced intracellular ROS content back to baseline nontransduced levels (Figure 5e) in part through notable increases in SOD activity (Figure 5f). Rather than blunting the cytokine signature of EDCs (Bonios et al., 2011), unbiased proteomic profiling revealed Mybl2 over‐expression altered production of factors within pathways known to promote angiogenesis (amphiregulin, endostatin) and reduce inflammation (IL‐1β, IL‐4, IL‐17a; Figure 5g and Figure S8). Unlike previous reports demonstrating EV transfer of engineered transcripts (Zomer et al., 2015), quantitative PCR screening of EVs from Mybl2 transduced cells failed to demonstrate appreciable increases in the Mybl2 transcript (Figure S9).

**Figure 6:**
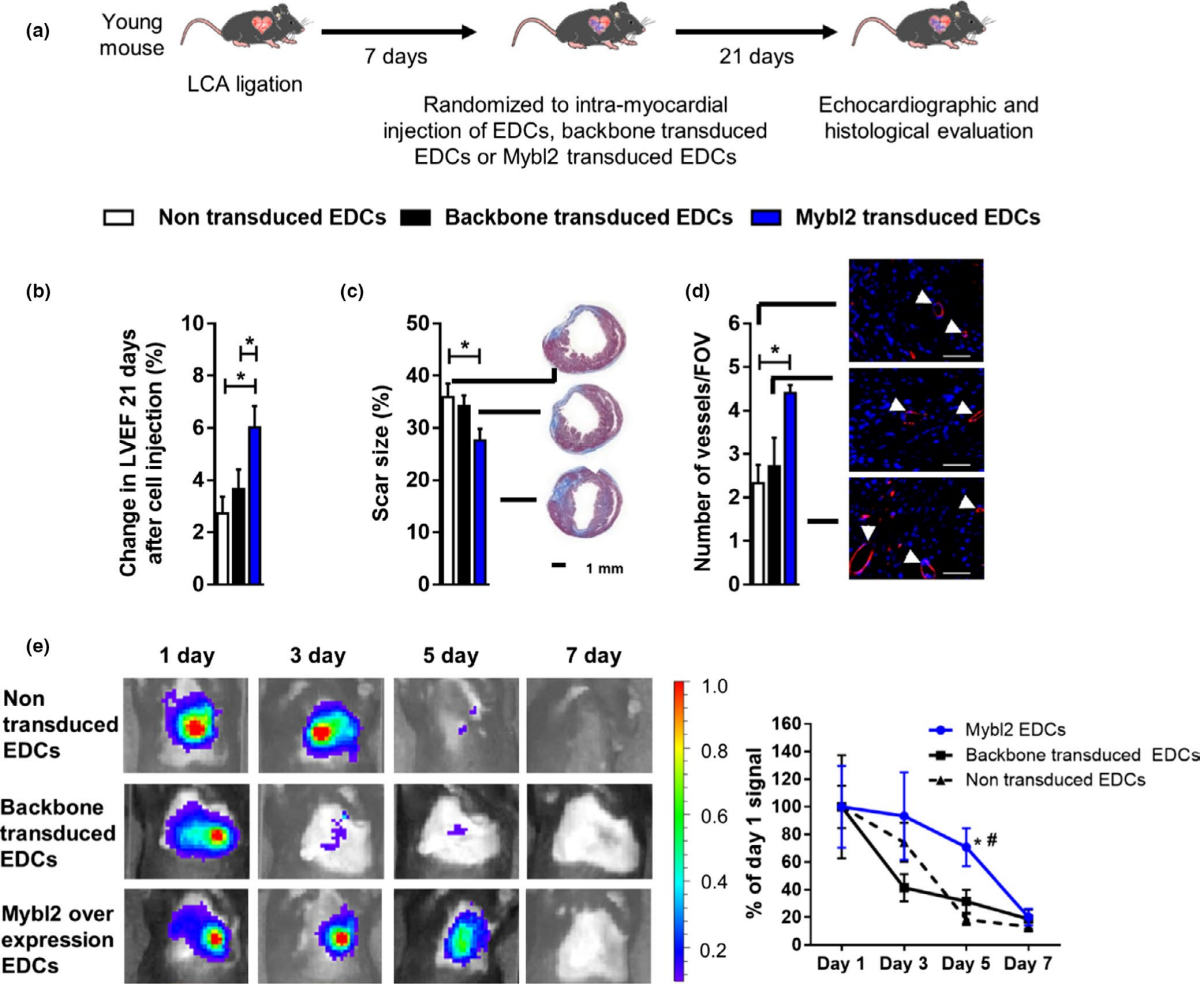
Mybl2 gene transfer effect on performance of EDCs in vivo. (a) A schematic overview of the experiment. (b) Improvement in heart function (∆EF %) at 3 weeks after EDC injection compared to baseline evaluation at Day 7 when Mybl2 over‐expressing EDC were transplanted into LCA ligated mouse heart (*n* = 9) (c) Scar size and representative pictures of Masson’s Trichrome staining (*n* = 5). (d) Quantification of vessel number in the border zone infarct area and representative images of isolectin B4 staining (*n* = 5). (e) Effect of Mybl2 over‐expression on EDC retention. Representative images of bioluminescence signal evaluated 1, 3, 5, and 7 days after EDC injection (right panel) and the percentage of retained cells determined by calculating the signal ratio to Day 1 (left panel; *n* = 6). Values are mean ± *SEM*. *p ≤ .05 as indicated; In E#*p* < .05 versus backbone transduced; *p < .05 versus nontransduced. EDCs, explant‐derived cells; LCA, left anterior coronary artery

The influence of Mybl2 on cell‐mediated repair of injured myocardium was investigated (Figure 6a). As shown in Figure 6b and Table S7, Mybl2 over‐expression increased the ability of transduced aged EDCs to promote myocardial function as compared to control or nontransduced aged EDCs. Similar effects were seen in final scar sizes (Figure 6c and Figure S10). Consistent with a role in cellular rejuvenation, the effect size seen in these parameters was equivalent to benefits seen after injection of EDCs from young noninfarcted donors (*p* = ns; Figure 2). In vivo vessel formation was measured following the observation that endostatin secretion was reduced by Mybl2 over‐expression. As shown in Figure 6d and Figures S11 and S12, transplant of Mybl2 over‐expressing EDCs increased vessel density to a level greater than what was observed after transplant of EDCs cultured from young noninfarcted donors (Figure 2d). Despite the alteration in inflammatory cytokine secretion, there was no appreciable difference in CD68+ cell density (data not shown). This finding may be attributed to the modest production of differentially expressed ILs from the nontransduced EDC or the fact that the post‐infarct‐inflammatory phase (macrophage recruitment) is essentially resolved by the time of heart collection. To probe whether the antisenescent effects of Mybl2 can be extended to in vivo cell retention, the effect of Mybl2 over‐expression was evaluated in a separate cohort of mice injected with EDCs transduced with luciferase. As shown in Figure 6e, bioluminescent signals diminished over time with Mybl2 treated EDCs demonstrating an appreciable difference, 5 days after injection (70.8 ± 13.7% relative to 31.6 ± 8.5 in control‐transduced or 18.5 ± 4 in nontransduced EDC treated mice). These data hint that the benefits observed after injection of EDCs genetically engineered to over‐express Mybl2 may be partially attributable to increased, albeit transient, persistence of transplanted cells.

## 3 DISCUSSION

Despite preclinical models reporting that heart‐derived cell therapy restores heart function after injury, favorable outcomes have not been reproduced by clinical trials and have led many to question the future of cell therapy (Capricor Inc, 2017; Rafatian & Davis, 2018). While many technical factors contribute (Kanda & Davis, 2017), the influence of common clinical modifiers on cell function are very rarely considered before transitioning to clinical trials. Previous work by our group has shown that accumulating medical comorbidities reduce the ability of heart‐derived cells to stimulate endogenous cardiac repair mechanisms (Mayfield et al., 2016, 2017; Molgat et al., 2014). In this study, we explored the effect of age and ischemia on cell function; two factors that a large number of patients in need of cell therapy possess. While some studies have evaluated the effects of these parameters on heart‐derived cell function, these studies have studied cells from patients with multiple medical comorbidities which cloud the straightforward interpretation of study outcomes (Cheng et al., 2014; Mishra et al., 2011; Nakamura et al., 2016). Interestingly, the only clinical studies of heart‐derived cells that have demonstrated consistently positive results have used autologous cells from pediatric donors (Ishigami et al., 2015, 2017; Tarui et al., 2015).

Initially, we found that advanced donor age reduced cell culture yields while ischemic injury improved cell formation from aged tissue. Although the cell source of EDCs within plated tissue is unclear, previous work has shown that, akin to epicardial progenitor cells (Smits, Dronkers, & Goumans, 2018), EDC growth depends on the epithelial‐to‐mesenchymal transition (EMT) within cardiac biopsies (Zakharova, Nural‐Guvener, & Gaballa, 2012). Consistent with this finding, our microarray data found that Notch signaling was downregulated in aged EDCs (Jagged1, Hes1, Hes7 and Numb1), while ICM increased Notch‐related molecules and, presumably, EMT‐mediated cell growth. Practically speaking, the proliferative effect of preexisting ischemic injury may be useful as collecting a clinical “cell dose” from aged patient suffering from ICM would not be complicated. In contrast, autologous cell growth might be challenging for samples from older patients with nonischemic heart failure. Ischemia also increased the number of senescent cells found in culture despite young mice cells having a greater ability to handle free radical insults. Mining our microarray data revealed that Sirtuin1 was downregulated in both aged and young mice after ischemia. Interestingly, inactivation of Sirtuin1 has been reported in cardiac tissue after ischemia/reperfusion (Tong et al., 2013) and this transcription factor epigenetically controls senescence (Ogryzko, Hirai, Russanova, Barbie, & Howard, 1996) by downregulation of frizzled‐related protein (Pruitt et al., 2006). In our study, frizzled‐related protein 1 was elevated after ischemia in both age‐groups, suggesting that Sirtuin1 inhibition resulted in the higher number of senescent cells after ischemia.

Ischemia altered the pro‐angiogenic potential of EDCs via changes in proliferin secretion. Surprisingly, these changes were not mirrored in vivo which may reflect the greater sensitivity of cultured HUVEC cells to pro‐angiogenic stimuli. Also, proliferin role may be tissue‐specific as one study about proliferin effect in cardiac tissue suggests a hypertrophic role for this factor (Dang et al., 2015).

The paracrine effect of EDCs was not limited to angiogenesis or recruitment of endogenous cells. EV RNA sequencing revealed that the mRNA content of vesicles secreted by aged ICM EDCs contained transcripts suspected to disrupt the cell cycle within target cells. Intriguingly, significant populations of altered miRNAs within aged ICM EDC‐secreted EVs are dysregulated in cardiomyopathic conditions (ischemia, hypertrophy and heart failure, or during exposure to toxic molecules) which suggests a pathological role (Li et al., 2016; Wang et al., 2017; Wei et al., 2015).

Our microarray data revealed that increased age also reduced the “self‐repair” ability of cells which was overcome by increasing Mybl2 expression. In vivo, this translated into functional and structural improvements to a level comparable with young EDC treatment. To the best of our knowledge, there is no study looking for the effect of Mybl2 in stem cell senescence and rejuvenation. Mybl2 over‐expression did not affect the proliferation rate in our cells (data not shown). Rather, it rejuvenated aged EDCs by reducing the number of senescent cells through reversing to a younger phenotype or eliminating some of the cells through apoptosis (data not shown). The benefits were also partly achieved thorough increased activity of antioxidant enzymes and greater retention of Mybl2 over‐expressing EDCs while forcing the adoption of a pro‐healing cytokine profile that stimulated endogenous repair to increase angiogenesis and improve heart function after injury. Reflecting on the physiological role of Mybl2 sheds important insight into the mechanism for these effects as Mybl2 (a) possesses antineoplasia properties and (b) has been shown to be a critical determinant of cell fate. The latter being particularly attractive as it identifies Mybl2 as a prototypical factor enabling targeted cellular rejuvenation (Clarke et al., 2013; Heinrichs et al., 2013; Mowla et al., 2014). To this point, Mybl2 has been identified as having a key role in maintaining genome integrity with no incidence of neoplastic conversion associated with Mybl2 over‐expression (Lorvellec et al., 2010; Tarasov et al., 2008; Yamauchi et al., 2008).

The marked reductions noted in cardiac functional improvement following cell transplantation into aged recipients can be extrapolated to Mybl2 over‐expressing EDCs. The reductions in functional improvement suggest that apart from donor age, recipient age is a large obstacle in conveying the benefits of EDC therapy and should be considered for better cell therapy outcomes. Therapeutic systemic interventions prior to cell transplantation may boost the cardio‐regenerative response and merit further investigation.

This study has several important limitations that include reliance on murine models. As such, the extent to which these findings may be extrapolated to human sourced cells or patients with ICM is unclear but, based on these promising results, deserves future attention. Although we found an increase in isolectin+ cells with a visible lumen and often saw evidence for blood cells within these sections, in the absence of clear perfusion data, we cannot be certain that the angiogenic effects of EDCs were truly functional. We also observed that Mybl2 over‐expression transiently prolonged engraftment, but the mechanisms are not clear and clearly deserve future attention using more sensitive techniques capable of relating the number of retained transplanted cells to changes increases in cell proliferation and/or resistance to apoptosis. This paper was intentionally positioned as a proof of principle study to determine whether Mybl2 could reverse the adverse combinatorial effects of advanced donor age and ischemia on cell function. For clinical purposes, exploring the effect of additional safety features will likely be warranted (i.e., nonintegrative and/or transient expression) but this is not a *sine qua non* as Mybl2 has many antineoplastic properties (Clarke et al., 2013; Heinrichs et al., 2013). Given that Mybl2 influences many downstream pathways and is regulated by many factors, more work is needed to identify the pathways enabling survival within damaged tissue to prevent cardiac dysfunction. Finally, the population studied represents an important limitation. We intentionally focused on 1‐year‐old donors and recipients which reflects the observed increase in clinical heart failure incidence (Bui, Horwich, & Fonarow, 2011) but does not the limits of mouse lifespan (≈2 years). Important follow‐up experiments are needed to appreciate if these findings can be extended to the extremes of aging.

To conclude, donor comorbidities help to define the reparative potential of autologous cellular therapeutics. Despite being direct contributors to the inevitable progression of heart failure, advanced age and ischemia are often overlooked in the preclinical evaluation of stem cell therapy candidates. Our findings, using a clinically relevant mouse model of advanced age and myocardial ischemia, identified a negative effect of age and ischemia on heart‐derived cell function associated with the activation of senescent pathways. We have identified Mybl2 as a prototypical antisenescent molecule capable of correcting cell cycle dysregulation and rejuvenating EDCs to prolong engraftment which promotes endogenous healing and improves cell treatment outcomes.

## 4 EXPERIMENTAL PROCEDURES

### 4.1 Study groups

Explant‐derived cells were obtained from aged (54 weeks) and young (8 weeks) female C57BL/6 mice. Mice were randomized to receive no intervention (control, Ctl; *n* = 88) or surgical ligation of the left anterior coronary artery (LCA; ischemic, ICM; *n* = 86). Four weeks after randomization, animals were sacrificed for EDC culture as previously described (Davis et al., 2010; Mayfield et al., 2016; Molgat et al., 2014). Cardiac explant‐derived stem cells (EDCs) sourced from the four groups of mice, young control donors, aged control donors, young donors with chronic ischemia, or aged donors with chronic ischemia, were used for further experimentation.

To evaluate the effect of Mybl2 over‐expression, EDCs sourced from aged donors with chronic ischemia underwent somatic gene transfer of Mybl2. Nontransduced EDCs and EDCs transduced by backbone lentivirus were served as controls. The effect of Mybl2 over‐expression on EDC function was evaluated 72 hr after EDC transduction using appropriate techniques.

### 4.2 Experimental animals, myocardial infarction, functional evaluation, and cell culture

C57BL/6 mice were handled in accordance with the Canadian Council on Animal Care Guide to the Care and Use of Experimental Animals under a protocol approved by the University of Ottawa Animal Care and Use Committee. For LCA ligation, animals were injected with buprenorphine (0.05 mg/kg; subcutaneous) 1 hr prior to surgery and twice daily thereafter for 3 days. During the ligation, mice were intubated and anesthetized using isoflurane (maintained at 2%–3%). Mice underwent echocardiographic (Vevo 770, VisualSonics) imaging 7, 21 and 28 days after LCA ligation (Figure S1). Cardiac dimensions and left ventricular ejection fraction (LVEF) were calculated from the parasternal images using standard techniques (Vevo 770 3.0.0, VisualSonics).

Four weeks after randomization, animals were sacrificed for EDC culture. Myocardial biopsies were minced, rinsed and digested (collagenase IV 1 mg/ml; Thermo Fisher) for 30 min at 37°C. After digestion, tissue fragments were plated on fibronectin (BD Biosciences)‐coated culture ware in standard media (Iscoves modified Dulbeccos medium) supplemented with 20% fetal bovine serum, 100 U/ml penicillin G, 100 µg/ml streptomycin, 2 mmol/l l‐glutamine, and 0.1 mmol/L 2‐mercaptoethanol at 37°C and 5% CO_2_ (all from Thermo Fisher) (Davis et al., 2010). The cell outgrowth that spontaneously migrates from plated tissue was collected for five times at 7‐day intervals for direct experimentation. The total number of harvested cells was counted with a Neubauer hemocytometer and normalized to heart weight. The phenotype of EDCs was profiled using flow cytometry (Guava easyCyte, Millipore) for CD90^+^ (A14726, Thermo Fisher), CD34+ (119309, Biolegend) and c‐Kit+ (sc‐168, Santa Cruz or A‐11034, Thermo Fisher) cell content.

Commercial mouse dermal fibroblasts (DF, cell biologics) and human umbilical vein endothelial cells (HUVECs, Lonza) were cultured according to the manufacturer’s directions. Bone‐marrow‐derived mononuclear cells were collected from femur and tibia of C57 mice and cultured for 7 days in EGM‐2 media (Lonza).

### 4.3 Evaluation of senescence

Bright‐field images of EDCs (Zeiss Axio ObserverA1) were used to trace cellular outlines for area, roundness and length/width ratios. Length/width ratios were calculated by dividing Feret/MinFeret measurements (Image J, NIH). A minimum of 30 cells originating from three different mice per cell line were evaluated. Plated EDCs underwent β‐galactosidase staining after overnight culture with SA‐β‐gal Detection Solution (KAA002, Millipore) followed by 4′,6‐diamidino‐2‐phenylindole (DAPI, Sigma) staining to count the total number of seeded cells. Six random fields were used to estimate the abundance of the β‐galactosidase. The percentage of senescent cells was calculated based on the number of β‐gal+ cells divided by the total number of cells. Expression of senescence markers was evaluated using enzyme‐linked immunosorbent expression of caveolin‐1 (MBS2025498, MyBioSource) and lamin‐1 (MBS2705839, MyBioSource). Finally, the proportion of senescent cells within murine noninfarcted tissue sections was evaluated using random field analysis after labeling with β‐galactosidase (ab203749, Abcam) and DAPI (*n* = 3 random fields/section).

### 4.4 Evaluation of antioxidant reserves

Glutathione peroxidase (GPx) and SOD activity were quantified using colorimetric assays according to the manufacturer’s directions (Cayman Chemical). Briefly, the supernatant from sonicated EDCs was collected for quantification of colorimeric absorption reflective of GPx or SOD activity. Reactive oxygen species content was measured using a fluorimetric assay (Abcam) according to the manufacturer’s directions. In brief, EDCs were incubated in culture with 2′, 7‐dichlorofluorescein diacetate for 1 hr followed by direct quantification of fluorescence intensity reflective of ROS burden. Treatment of EDCs with tert‐butyl hydrogen peroxide (50 μM) for 1 hr was used as a positive control.

### 4.5 Characterization of extracellular vesicles secreted by EDCs and miRNA sequencing

Extracellular vesicles were collected from EDCs cultured in hypoxic (1% oxygen) and low serum (1% exosome depleted serum; System Bioscience). Conditioned media was centrifuged at 10,000 *g* for 30 min to remove cellular debris prior to centrifugation at  100,000 *g* for 3 hours to remove the large particles/vesicles. This supernatant was then centrifuged at 28,000 rpm for 3 hr to pellet extracellular vesicles (L8‐70M, Beckman). The isolated vesicles were used for characterization measurements, total RNA, and miRNA extraction. EV surface marker expression was quantified using enzyme‐linked immunosorbent expression of CD9 (MyBioSource, MBS944415), FLOT‐1 (MyBioSource, MBS7230473), and HSP gp96 (Novus Biologicals, NBP2‐76452). The total vesicles pelleted from 6 ml of EDC conditioned media were resuspended in PBS for quantification and evaluation of the particles size distribution by NanoSight (LM10; Malvern Instruments). A total of 55 ml of conditioned media was used to extract total RNA or miRNA. The extracellular vesicles were dissolved in QIAzol lysis reagent (Qiagen), and RNA was extracted according to the manufacturer’s protocol (RNeasy or miRNeasy Mini Kits, Qiagen). Mybl2 transcript expression within EVs was evaluated using qPCR for commercial primers (Integrated DNA Technologies). The quality and size of miRNA samples were evaluated by Fragment Analyzer (Advanced Analytical) and Qubit assay (Thermo Fisher Scientific) prior to library generation. The miRNA library was generated using Small RNA‐Seq Library Prep Kit (Lexogen). The quantity and quality of the resultant DNA product were assessed by Qubit and Fragment Analyzer, respectively. Twelve samples with different indexes were run on MiniSeq system (Illumina) for a total of 25 million reads. Sequencing was performed with high‐output module and was single‐end 1 x 75 base.

To analyze the sequencing data, after trimming for adaptors, the quality score and length of the sequences were assed. Using Burrows‐Wheeler Aligner algorithm, short nucleotide sequences were mapped to the reference genome (Mus_musculus. GRCm38—v94), and using DESeq2 software, the sequences with differential expression was identified. Further investigation on differentially secreted RNA was performed using miRDB (MicroRNA Target Prediction And Functional Study Database), PANTHER gene list analysis, and DAVID Functional Annotation tool.

### 4.6 Microarray analysis

Total RNA was extracted using the RNeasy Mini Kit (Qiagen) as per the manufacturer’s protocol, and quantity/quality was assessed using NanoDrop 2000 and 2100‐Bioanalyzer system (Agilent Technologies). The same RNA samples were used for subsequent qPCR validation. Amplified cRNA was streptavidin‐labeled, fragmented, and hybridized to Affymetrix Mouse Gene 2.0 ST arrays as recommended by the manufacturer (Affymetrix, Santa Clara). Expression data were analyzed by statistical analysis of microarray software, with a calculated median FDR of = 5%. Ingenuity pathway analysis program was used for comparison of canonical pathways between groups. Genes with 1.5‐fold different expression rate were used for hierarchical clustering (Array Star v12, DNASTAR). Among these genes, those that were related to the senescence pathway or the mitochondrial function were distinguished. Microarray data were validated by qPCR of five random genes (Figure S7) with different levels of mRNA expression (Ppp1r16b, Tnf, Cxcr4, Rgs1, and Sirt6) using appropriate primers obtained from IDT.

### 4.7 Intramyocardial cell injection, functional evaluation, and in vivo assays

A separate cohort of aged and young female mice were randomized 7 days after LCA ligation to receive vehicle or 100,000 EDCs from aged or young mice with and without a history of myocardial infarction as a divided dose between the cardiac apex and lateral border zone (Mayfield et al., 2016; Molgat et al., 2014). This period of time that is clinically relevant and allows the resolution of the inflammatory phase. Transthoracic intramyocardial injection was performed using echocardiographic guidance to confirm cells were injected into the myocardium (baseline). Twenty‐one days after cell injection, the effect of cell therapy was evaluated using echocardiography for LVEF and chamber dimensions. After the final echocardiogram, mice were sacrificed, and hearts were excised for histological evaluation. The hearts were fixed with 4% paraformaldehyde, embedded in paraffin, and sectioned. Four sections per heart were stained by Masson’s trichrome (Thermo Fisher) to measure scar size. Scar size was calculated from Masson’s trichrome stained sections by measuring the percentage of blue pixels in the infarct region to the overall heart pixels using ImageJ software. A separate set of sections were deparaffinized and blocked prior to staining for isolectin B4 (B‐1205, Vector Laboratories), alpha smooth muscle acting (ab5694, Abcam), or von Willebrand factor (11778‐1‐AP, Proteintech). After DAPI counterstaining, the number of vessels per field of view within the infarct/border zone was assessed by fluorescent microscopy.

### 4.8 Statistical analysis

All data are expressed as mean ± *SEM*. Normalcy and variance of data were evaluated prior to statistical analysis. Data were analyzed by one‐way ANOVA multiple comparison with Sidak’s correction or Dunnett’s corrected *t* test (Prism v6). All *p* values are two‐sided, and *p* ≤ .05 was considered statistically significant.

## CONFLICT OF INTEREST

None of the authors have any conflicts to disclose.

## AUTHOR CONTRIBUTIONS

G.R., E.J.S., and D.R.D. conceptualized the study; G.R., E.J.S., and D.R.D. involved in methodology planning; G.R., and D.R.D. involved in formal analysis; G.R., M.K., A.S.D.M., and R.S. investigated the study; G.R., and D.R.D. wrote the original draft of the manuscript; G.R., M.K., A.S.D.M., E.J.S., and D.R.D. reviewed and edited the final version of the manuscript; E.J.S. and D.R.D. involved in resource management; E.J.S. and D.R.D. supervised the study; and D.R.D. acquired funds for the study.

## Supporting information

Supplementary Material

Supplementary Material

## Data Availability

The Sequencing and Microarray data that support the findings of this study are openly available in Mendeley at http://doi:10.17632/js74ttpdjv.1
