# Supplementary material for: Mybl2 rejuvenates heart explant‐derived cells from aged donors after myocardial infarction
Source: Aging Cell. 2020 Jun 19;19(7):e13174. doi: 10.1111/acel.13174 (PMC7433005; doi:10.1111/acel.13174)
Supplement: Supplementary file 1 — Supplementary Material [file ACEL-19-e13174-s001.docx]

Supporting Information

Mybl2 Rejuvenates Heart Explant-Derived Cells from Aged Donors after Myocardial Infarction

Ghazaleh Rafatian PhD, Maryam Kamkar PhD, Sandrine Parent, Connor Michie, Yousef Risha MSc, André SD Molgat PhD, Richard Seymour, Erik J Suuronen PhD, and Darryl R Davis MD


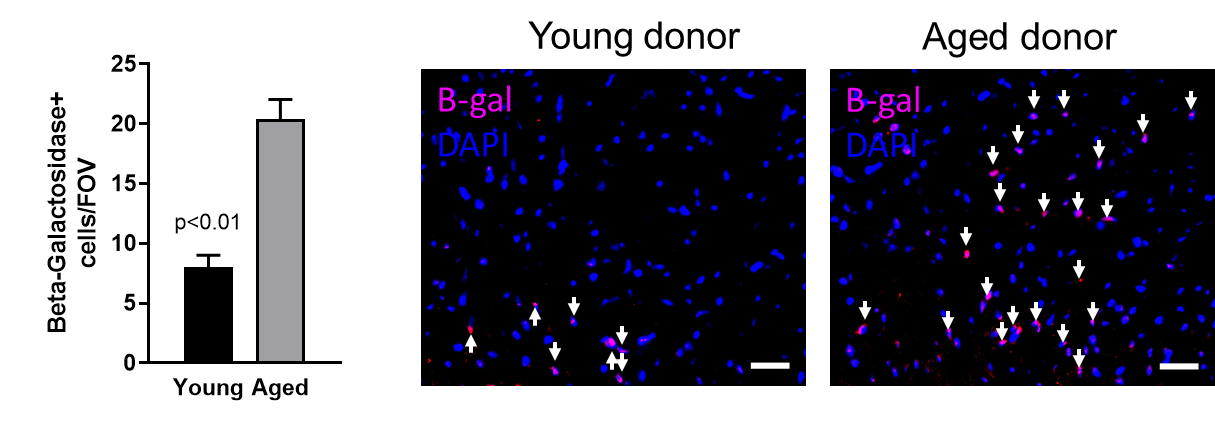


Supplementary Figure 1. Representative images of cardiac sections and random field analysis for beta-galactosidase demonstrating increased expression in aged and young mice (n=5). Scale bar 50 μm.


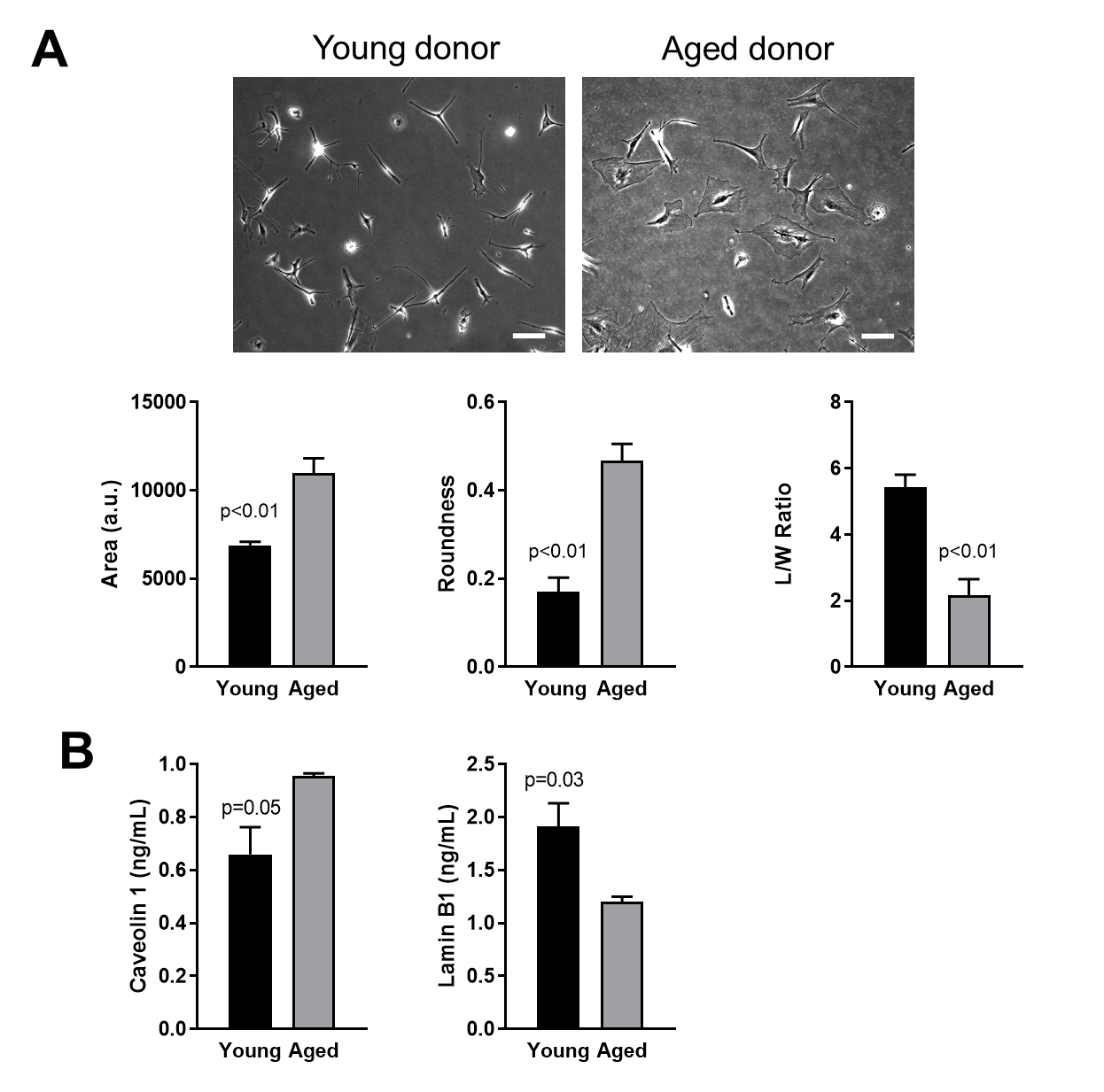


Supplementary Figure 2. Expression of senescence markers in EDCs from aged and young donors. (A) Representative images and quantification of EDCs cell size from aged and young donors. Random field analysis of individual plated cells for measures of cell area, roundness and length to width (L/W) ratio (n = 3). Scale bar 200 µm. (B) Enzyme-linked immunoassay analysis of Caveolin 1 and Lamin B1 expression within aged and young EDCs (n=3). Values are mean ± SEM.

Supplementary Figure 3. Cardiac remodeling after experimental myocardial infarction. Old and young mice underwent LCA ligation and had serial echocardiograms performed 7, 21 and 28 days later (n=17/group).


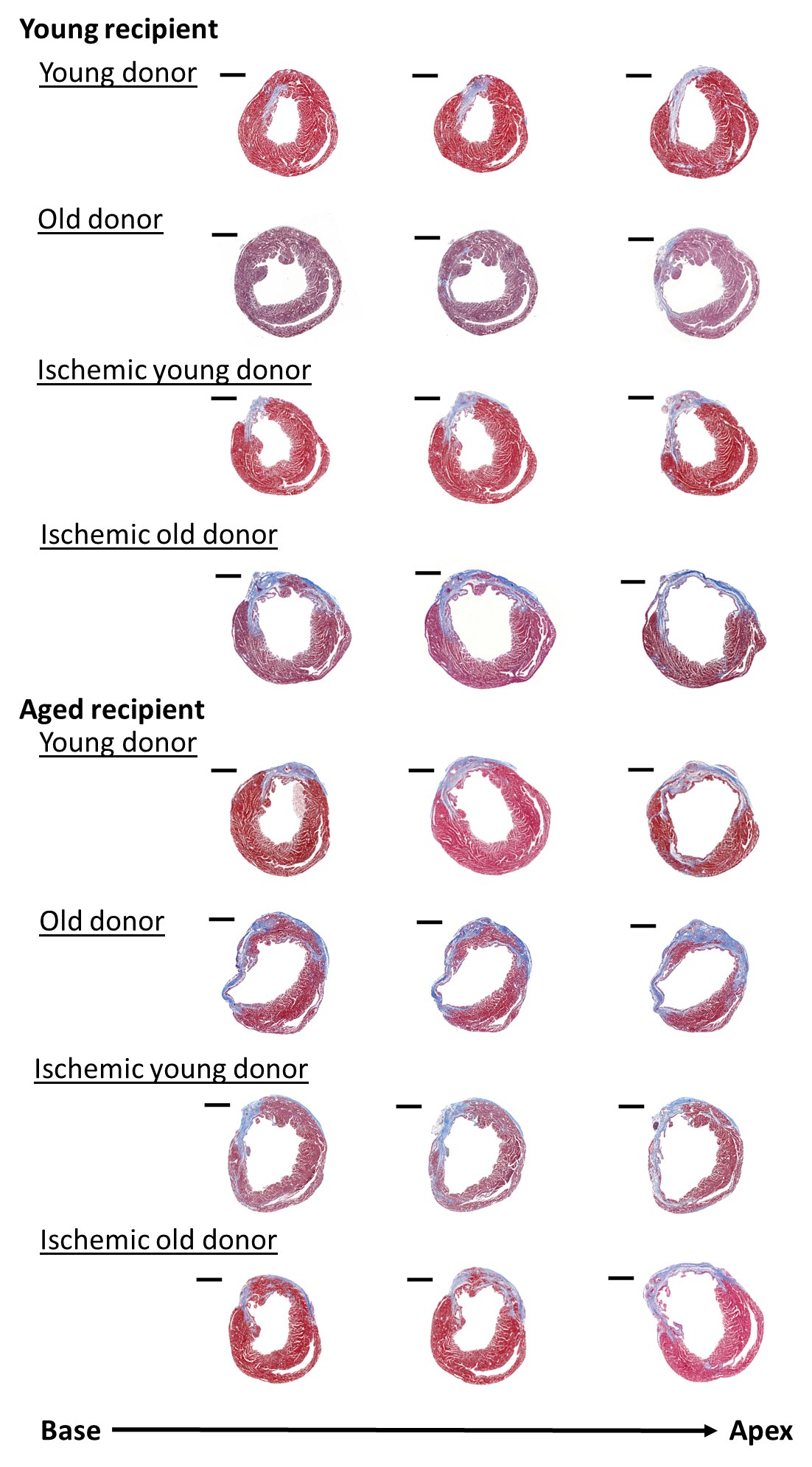


Supplementary Figure 4. Representative images of Masson’s Trichrome stained histological sections from aged and young mice that received EDCs from aged or young donors with or without a history of ICM. To endure consistency, base, med-cavitary and apical sections were taken 20, 40 and 60 μm from the ligature, respectively. Scale bar 1000 μm. Values are mean ± SEM.

Supplementary Figure 5. Profiling of secreted angiogenic cytokines in EDCs and the negative control DF cells (n=3). *p≤0.05 as indicated; # p≤0.05 in all EDC groups compared to DF cells. ICM, ischemic. Values are mean ± SEM.

Supplementary Figure 6. Enzyme linked immunoassay analysis of markers typically enriched (CD9, FLOT-1) or depleted (gp96) in EDC EVs (n=6). Values are mean ± SEM.

A

B

Supplementary Figure 7. Microarray data validation. (A) Five genes were selected for qPCR. The correlation ratio validated the accuracy of microarray data (n=3). (B) qPCR for selected transcriptome, Mybl2 gene, for modification based on microarray data (n=3). ICM, ischemic. Values are mean ± SEM.

Supplementary Figure 8. Profiling of mouse secreted cytokines after Mybl2 over-expression. 111 cytokines were quantified within conditioned media (n=3). There was no difference between control-transduced and non-transduced cytokine secretion. The data for the two control groups are combined for this picture. *p≤0.05 Mybl2 transduced vs. both controls. Values are mean ± SEM.

Supplementary Figure 9. Expression of the MYBL2 transcript within EDCs and EVs from aged donors transduced with MYBL2.


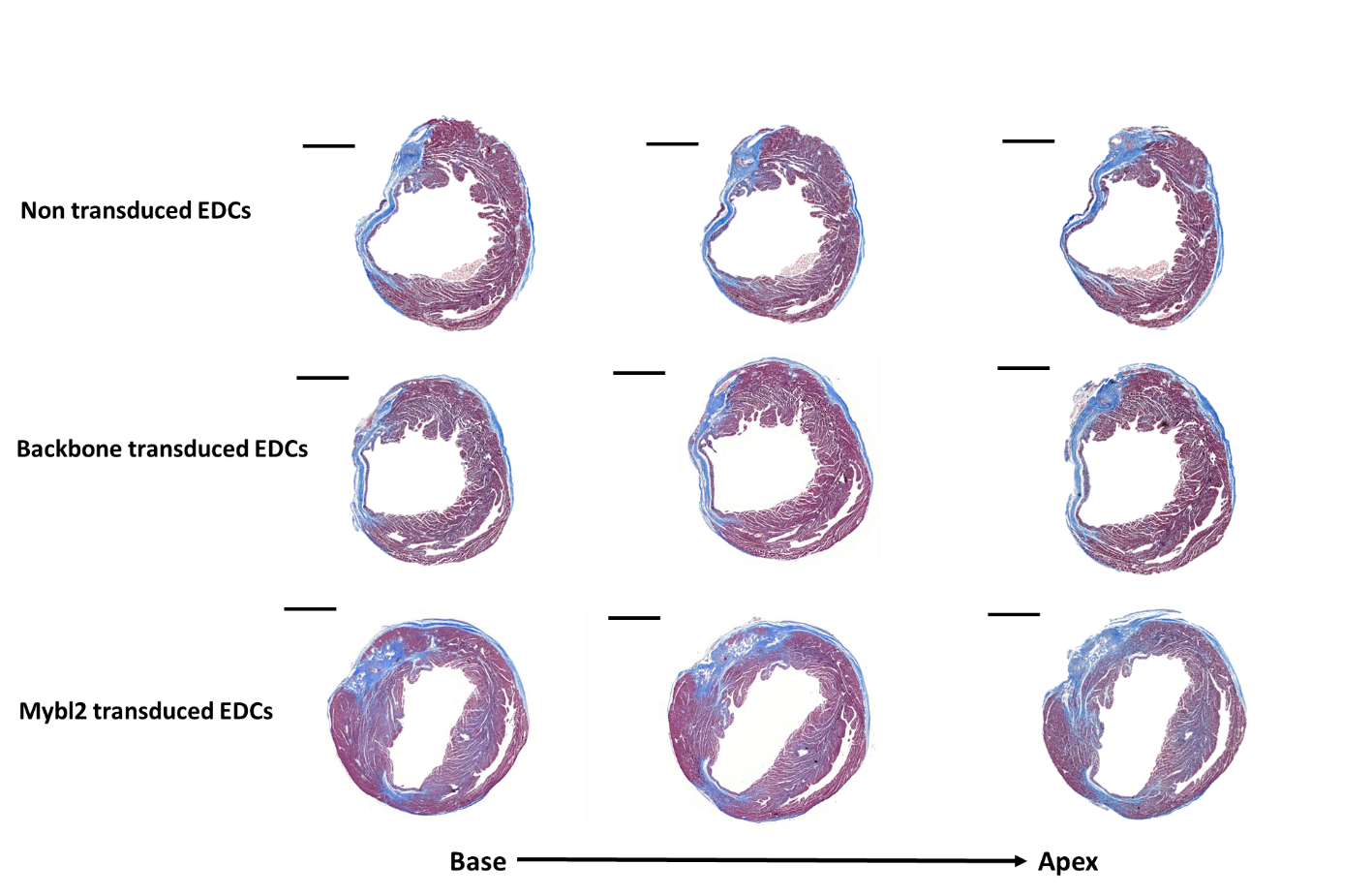


Supplementary Figure 10. Representative images of Masson’s Trichrome stained histological sections from mice that received EDCs from aged donors transduced with MYBL2, transduced with the backbone vector or non-tansduced. To endure consistency, base, med-cavitary and apical sections were taken 20, 40 and 60 μm from the ligature, respectively. Scale bar 1000 μm. Values are mean ± SEM.


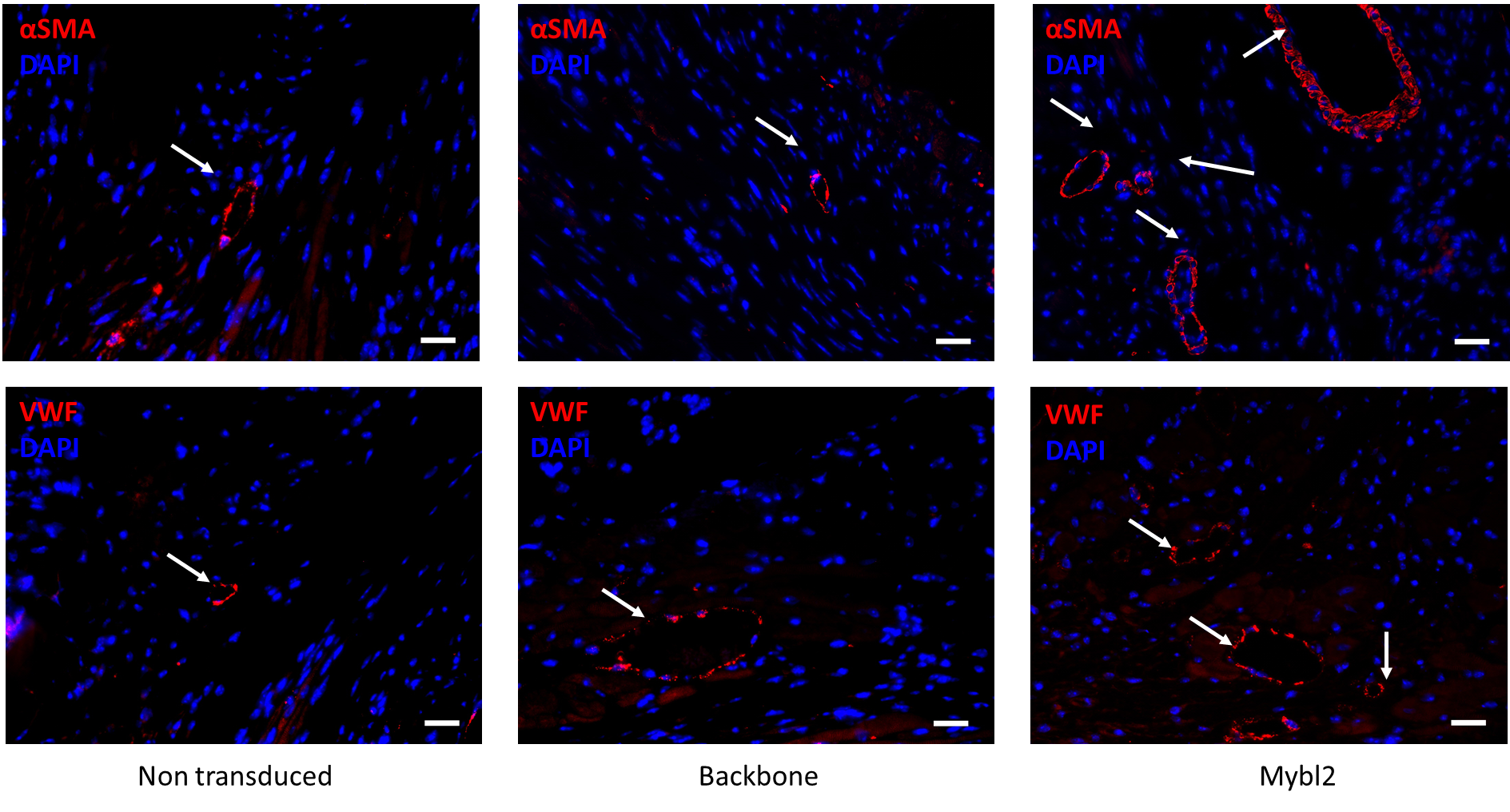


Supplementary Figure 11. Representative immunohistological images of capillary (vWF+) and arteriolar (alpha-SMA+) densities. Scale bar 50 m.

Supplementary Figure 12. Effect of Mybl2 on capillary (vWF+) and arteriolar (alpha-SMA+) densities. Values are mean ± SEM. *p≤0.05 vs. backbone or non-transduced EDCs.

|  | Young donor | | Old donor | |
| --- | --- | --- | --- | --- |
| Days post MI | 7 | 28 | 7 | 28 |
| EDV | 55.9±4 | 68.3±4.1 | 85.4±4.7* | 91±4.2* |
| ESV | 38.3±3.6 | 49.1±4 | 60.8±3.9* | 66.7±3.7* |
| SV | 17.6±0.9 | 19.2±0.8 | 24.5±1.3* | 24.3±1.3* |
| LVEF (%) | 33.1±1.9 | 29.7±1.7 | 29.3±1.4 | 27±1.4 |
| FAC (%) | 20.3±1.1 | 18.3±1.2 | 17.4±1 | 16.2±1 |

Supplementary table 1. Echocardiographic parameters after LCA ligation. Values are mean ± SEM. *p≤0.05 vs. PBS group; # p≤0.05 day 7 vs. day 28; +p≤0.05 young vs. old; EDV: End diastolic volume; ESV: End systolic volume; SV: Stroke volume; LVEF: Left ventricular ejection fraction; FAC: Fractional area shortening.

| Young Recipient | | | | | | | | | | |
| --- | --- | --- | --- | --- | --- | --- | --- | --- | --- | --- |
| Donor: | PBS  control | | Young mouse  no ischemia | | Old mouse  no ischemia | | Young mouse  with ischemia | | Old mouse  with ischemia | |
| Days post MI | 7 | 28 | 7 | 28 | 7 | 28 | 7 | 28 | 7 | 28 |
| EDV | 64±1 | 76±4# | 57±4 | 72±4# | 60±4 | 69±2 | 65±3 | 76±3# | 68±4 | 79±8 |
| ESV | 44±2 | 55±4# | 41±4 | 46±3 | 42±3 | 45±2 | 48±3 | 51±3 | 48±4 | 55±7 |
| SV | 20±1 | 21±2 | 16±1 | 25±1#* | 18±1 | 24±1# | 17±1 | 26±1#* | 19±1 | 24±1# |
| LVEF (%) | 32±2 | 28±3 | 28±2 | 36±2#* | 30±1 | 35±1#* | 27±2 | 34±2# | 28±2 | 33±2 |
| FAC (%) | 19±1 | 17±2 | 16±1 | 23±1#* | 17±1 | 22±1#* | 16±1 | 21±2# | 16±1 | 19±2 |

| Aged Recipient | | | | | | | | | | | |
| --- | --- | --- | --- | --- | --- | --- | --- | --- | --- | --- | --- |
| Donor: | PBS  control | | Young mouse  no ischemia | | Old mouse  no ischemia | | Young mouse  with ischemia | | Old mouse  with ischemia | |  |
| Days post MI | 7 | 28 | 7 | 28 | 7 | 28 | 7 | 28 | 7 | 28 |  |
| EDV | 113±11 | 103±9 | 93±5 | 93±4 | 94±6 | 88±4 | 108±9+ | 107±5 | 86±3 | 87±4 |  |
| ESV | 79±9 | 74±8 | 64±4 | 69±4 | 70±5 | 66±4 | 79±8+ | 81±4 | 60±3 | 61±4 |  |
| SV | 35±3 | 29±2 | 30±2 | 24±2+ | 24±1 | 22±1 | 29±2 | 26±2 | 27±2 | 26±1 |  |
| LVEF (%) | 31±2 | 29±2 | 32±2 | 26±2#+ | 27±1 | 25±2 | 28±1 | 24±2 | 30±2 | 29±1 |  |
| FAC (%) | 18±2 | 18±1 | 19±2 | 16±1 | 16±1 | 14±1 | 16±1 | 14±1 | 18±1 | 17±1 |  |

Supplementary table 2. Echocardiographic parameters after EDC therapy at day 7 post infarction when the EDCs were injected and 28 days after infarction (21 day after EDC therapy). Values are mean ± SEM. *p≤0.05 vs. PBS group; # p≤0.05 day 7 vs. day 28; +p≤0.05 young vs. old; EDV: End diastolic volume; ESV: End systolic volume; SV: Stroke volume; LVEF: Left ventricular ejection fraction; FAC: Fractional area shortening.

Supplementary table 3. List of small RNA with differential secretion within extracellular vesicles (p≤0.01).

| miRNA | Total predicted targets | Angiogenesis | Apoptosis | Cell cycle | Inflammation | Role based on literature |
| --- | --- | --- | --- | --- | --- | --- |
| mmu-miR-5110 | 773 | 9 | 4 | 2 | 11 | Correlates with high fat diet |
| mmu-miR-5106 | 147 | 2 |  |  | 2 |  |
| mmu-miR-5124a | 189 |  | 2 | 1 | 3 |  |
| mmu-miR-5119 | 170 | 3 | 2 |  | 1 |  |
| mmu-miR-5112 | 396 | 7 | 5 |  | 3 | Increases in viral myocarditis |
| mmu-miR-5108 | 163 |  | 2 | 1 | 2 |  |
| mmu-miR-2183 | 143 | 3 | 1 | 1 |  | Negatively correlates with GATA4 |
| mmu-miR-1957b | 379 | 9 | 4 | 9 |  | Proinflammatory |
| mmu-miR-100-5p | 27 | 2 |  |  |  | Down regulates in cells exposed to benzen pollutant |
| mmu-miR-99b-5p | 27 |  |  |  |  | Correlates with ischemia |
| mmu-miR-541-5p | 133 |  |  |  | 3 |  |
| mmu-miR-99a-5p | 27 |  |  |  |  | Attenuates cardiac hypertrophy |
| mmu-miR-10a-5p | 214 | 3 | 5 |  | 3 |  |
| mmu-miR-709 | 1024 | 24 | 8 | 1 | 19 | Correlates with heart failure induced by pressure overload |
| mmu-miR-2137 | 7 |  |  |  |  | Promotes inflammation |
| mmu-miR-151-5p | 32 |  |  |  |  |  |
| mmu-miR-184-3p | 39 | 1 |  |  |  | Anti-apoptotic |

Supplementary table 4. miRNAs with differential secretion within extracellular vesicles, their total number of predicted target genes, the number of target genes with role in angiogenesis, apoptosis, cell cycle and inflammation, and their role in cardiac tissue if reported in the literature.

Supplementary table 5. List of all RNAs with differential secretion within extracellular vesicles (p≤0.01).

Supplementary table 6. Microarray analysis. (A) list of upstream regulators predicted to be activated or inhibited by Ingenuity Pathway Analysis program and (B) list of genes with more than 1.5-fold differential expression (in EDCs sourced from ischemic old mice relative to ischemic young mice).

| Donor: | Mybl2 transduced | | Backbone transduced | | Non-transduced | |
| --- | --- | --- | --- | --- | --- | --- |
| Days post MI | 7 | 28 | 7 | 28 | 7 | 28 |
| EDV | 50.6±2.4 | 60.7±1.8# | 48.6±3.2 | 62.4±3.6# | 48.5±2.5 | 60.5±2.7# |
| ESV | 33.7±1.9 | 36.8±1.7 | 33.2±2.8 | 40.2±3 | 32.2±2.2 | 38.6±2.8 |
| SV | 16.9±1 | 23.9±1.1# | 15.4±0.7 | 22.3±0.9# | 16.3±0.8 | 21.9±0.7# |
| LVEF (%) | 33.5±1.4 | 39.5±1.7# | 32.5±1.6 | 36.2±1.4 | 33.9±1.7 | 36.7±1.8# |
| FAC (%) | 20.3±1 | 25.3±1.2# | 18.6±1.1 | 22.5±0.9# | 20.6±1.1 | 21.8±1.3# |

Supplementary table 7. Echocardiographic parameters after transgenic EDC injection at day 7 post infarction when the EDCs were injected and 28 days after infarction (21 day after EDC therapy). Values are mean ± SEM.; # p≤0.05 day 7 vs day 28; EDV: End diastolic volume; ESV: End systolic volume; SV: Stroke volume; LVEF: Left ventricular ejection fraction; FAC: Fractional area shortening.
