# Supplementary material for: Mybl2 rejuvenates heart explant‐derived cells from aged donors after myocardial infarction
Source: Aging Cell. 2020 Jun 19;19(7):e13174. doi: 10.1111/acel.13174 (PMC7433005; doi:10.1111/acel.13174)
Supplement: Supplementary file 2 — Supplementary Material [file ACEL-19-e13174-s002.docx]

**Supporting Information**

**Mybl2 Rejuvenates Heart Explant-Derived Cells from Aged Donors after Myocardial Infarction**

Ghazaleh Rafatian PhD, Maryam Kamkar PhD, Sandrine Parent, Connor Michie, Yousef Risha MSc, André SD Molgat PhD, Richard Seymour, Erik J Suuronen PhD, and Darryl R Davis MD

**Paracrine profiling of conditioned media**

Conditioned media was generated after 48 hours of culture within hypoxic (1% O_2_) low serum (1% FBS) media (IMDM, 100 U/ml penicillin G, 100 ug/ml streptomycin, 2 mmol/l L-glutamine and 0.1 mmol/l 2-mercaptoethanol) to simulate the ischemic transplant environment. Commercial enzyme-linked immunosorbent assays (ELISAs) quantified the abundance of angiogenin (MBS160147, MyBiosource), angiopoietin-1 (MBS163055, MyBiosource), hepatocyte growth factor (HGF, MHG00, RD Systems), interleukin-6 (IL-6, M6000B, RD Systems) and vascular endothelial growth factor (VEGF, MMV00, RD Systems). The angiogenic cytokine profile of EDC-conditioned media was surveyed using the Proteome Profiler Mouse Angiogenesis Array, according to the manufacturer's instructions (ARY015, R&D Systems) with relative abundance compared using ImageJ software analysis. A second proteomic array of 111 mouse cytokines (Proteome Profiler Mouse XL Cytokine Array; ARY028, R&D Systems) was used to evaluate the effect of lentiviral overexpression Mybl2 or transduction (backbone) on cytokine secretion within conditioned media.

**Angiogenic and migratory capability of cardiac explant-derived stem cell conditioned media**

The capacity of EDC conditioned media to promote *in vitro* angiogenesis was evaluated after exposure of HUVECs within a cytokine-depleted matrigel assay (ECM625, Millipore; µ-Slide Angiogenesis, ibidi) (Latham et al., 2013). After 16 hours of incubation, tube formation was quantified (NeuronJ ImageJ plugin, NIH). Tubule length for each sample was averaged from 3 well replicates To evaluate the influence of proliferin on angiogenesis, tubule formation assay was conducted after depleting Proliferin by incubating conditioned media with goat anti-mouse proliferin antibody (sc-47347 N-14; Santa Cruz Biotechnology) or goat IgG isotype control (ab-37373, Abcam) at 4 °C for 2 h prior to magnetic separation using protein A/G magnetic beads (88802, Life Technologies) for 3 h at 4 °C (Yang et al., 2012).

The effects of EDC-conditioned media on BMDC migration was evaluated using fibronectin-coated transwell plates (24 wells, 3.0 μm pores; Corning) with 1 x 10^5^ BMDCs seeded into the upper well within serum-free IMDM and EDC-conditioned media placed in the bottom well. Eighteen hours later Cells that had successfully migrated through the polycarbonate membrane were fixed with 4% paraformaldehyde and stained with 4',6-diamidino-2-phenylindole (DAPI, Sigma-Aldrich). To evaluate the effect of IL-6 on migration, conditioned media were incubated with rat anti-mouse IL-6 antibody (16-70611-85; eBioscience) or rat IgG1 isotype control (400402, Biolegend) at 4 °C for 2 h and then incubated with anti-rat Dynabeads (11035, Life Technologies) for 3 h at 4 °C. Migration assay was performed with IL-6 depleted conditioned media followed by quantification of the total number of cells in six random fields of view.

**Resistance to apoptosis**

The capacity of EDCs to withstand programmed cell death was assessed after 48 hours of exposure to hypoxic low serum conditions. Apoptosis was evaluated by staining the cells with 7-Amino-Actinomycin D (7-AAD) and Annexin V-PE (559763, BD Biosciences) for 15 minutes at room temperature. Flow cytometry was used to quantify the percentage of early and late apoptotic EDCs. Cells that expressed annexin V but not 7-AAD were categorized as early apoptotic while cells expressing both markers were late apoptotic.

**Plasmid constructs**

Commercial 3rd generation packaging plasmids i.e. RSV-REV, Rev Response Element (RRE) and VSV-G plasmid (addgene), 2nd generation packaging plasmids (LV003, abmgood), green fluorescent protein (GFP) expression vector (12154, addgene) and Mybl2 lentiviral vector (pLenti-GIII-CMV-GFP-2A-Puro; LV437429, Applied Biological Materials-abm) were purchased. Also, custom made firefly luciferase reporter plasmid (pCDH-EF1-FLuc-IRES-Puro, Dr. Duncan Stewart lab gift) and custom-made Backbone control vector were used in this study.

**Viral Packaging and cell transduction**

Lentiviruses were produced using HEK 293T cells in about 70% confluence after transfection with transfection reagent (Lipofectamine 2000, Thermo Fisher Scientific), packaging plasmids and the plasmid containing the gene of interest, with 48 and 72 hours later harvests followed by concentration (Lenti-X concentrator; Clontech) according to the manufacturer’s instruction. To transduce EDCs, cells were treated with 0.8 µg/ml polybrene (Sigma) for 10min prior to transduction with desired lentivirus. EDCs were then allowed to recover for at least 48 hours before further experimentation on them.

**Mybl2 over-expression confirmation**

Mybl2 and its target genes expression was confirmed at the RNA level. Mybl2, Cyclin B, CDK1 and FoxM1 mRNA was quantified 48 hours after lentiviral transduction using qPCR detection of mRNA (PrimeTime Predesigned qPCR probes, Integrated DNA Technologies). At the protein level, Mybl2 was evaluated 48 hours after transduction using CytoGlow B-Myb Cell-Based ELISA detection Kit (CB5077, Assay Biotech). The effect of Mybl2 over-expression on EDC function was evaluated 72 hours after EDC transduction using appropriate techniques.

**Measurement of short-term retention**

EDCs transduced with Mybl2 or Backbone were double transduced with Luciferase reporter gene. Seven days after LCA ligation, mice were randomized to receive 100,000 EDCs split into 2 doses delivered at the cardiac apex and lateral border zone using echocardiographic guidance. Luminescence by retained cells was evaluated after subcutaneous injection of 150 mg/kg of D-Luciferin (LUCNA, Gold Biotechnology) using serial whole-body imaging (IVIS spectrum in vivo imaging system, PerkinElmer) for 1 hour after injection to identify peak luminescence 1,3, 5 and 7 days after cell injection.
